# *Tristemma hirtum* and Five Other Cameroonian Edible Plants with Weak or No Antibacterial Effects Modulate the Activities of Antibiotics against Gram-Negative Multidrug-Resistant Phenotypes

**DOI:** 10.1155/2018/7651482

**Published:** 2018-03-22

**Authors:** Gaëlle S. Nguenang, Armelle T. Mbaveng, Aimé G. Fankam, Hermione T. Manekeng, Paul Nayim, Brice E. N. Wamba, Victor Kuete

**Affiliations:** Department of Biochemistry, Faculty of Science, University of Dschang, Dschang, Cameroon

## Abstract

In order to contribute to the fight against infectious diseases, the* in vitro* antibacterial activity and the antibiotic-potentiating effects of* Tristemma hirtum* and five other Cameroonian edible plants have been evaluated against Gram-negative multidrug-resistant (MDR) phenotypes. The microdilution method was used to evaluate the bacterial susceptibility of the extracts and their combination to common antibiotics. The phytochemical screening of the extracts was carried out according to standard methods. Phytochemical analysis of the extracts revealed the presence of alkaloids, triterpenes, steroids, and polyphenols, including flavonoids in most of the tested extracts. The entire tested extracts showed moderate (512 *μ*g/mL ≤ MIC ≤ 2048 *μ*g/mL) to weak (MIC > 2048 *μ*g/mL) antibacterial activities against the tested bacteria. Furthermore, extracts of leaf of* Tristemma hirtum* and pericarp*s* of* Raphia hookeri* (at their MIC/2 and MIC/4) strongly potentiated the activities of all antibiotics used in the study, especially those of chloramphenicol (CHL), ciprofloxacin (CIP), kanamycin (KAN), and tetracycline (TET) against 70% (7/10) to 100% (10/10) of the tested MDR bacteria, with the modulating factors ranging from 2 to 128. The results of this study suggest that extracts from leaves of* Tristemma hirtum* and pericarps of* Raphia hookeri* can be sources of plant-derived products with antibiotic modifying activity.

## 1. Introduction

During the last decade, the number of multidrug-resistant (MDR) pathogenic bacteria has dramatically increased all over the world [1–3]. The burden of MDR Gram-negative bacteria infections is particularly concerning because such bacteria are demonstrating resistance to nearly all currently licensed antibiotics [2, 4]. As a consequence, inadequate empirical antibacterial therapy of severe infections caused by MDR Enterobacteriaceae as well as* Pseudomonas aeruginosa* and* Acinetobacter baumannii* has been associated with increased morbidity and mortality [5, 6]. In this alarming scenario, the discovery of novel drugs that could provide clinical efficacy against MDR Gram-negative pathogens remains one of the keys to successfully overcome the tide of resistance [1, 4]. Targeting MDR systems as efflux in antibiotic resistant pathogens seems to be one of the most important existing strategies [4, 7]. For example, the drug combination ceftolozane-tazobactam has shown* in vitro* activity against selected MDR Gram-negative pathogens, including* P. aeruginosa* [8]. This combination is effective due to the ability of ceftolozane to evade multiple resistance mechanisms including efflux pumps, reduced uptake through porin channels, and modification of penicillin-binding proteins [9]. In the same goal, many other studies have been done and some are ongoing. Plants constitute an undeniable source for the discovery of new antibacterials acting directly as bacterial growth inhibitors or as antibiotic modulators [10–12]. Previous works showed that edible plants have excellent antibacterial properties and could also act as antibiotic modulators [13–18]. In the continuous contribution to the fight against infections due to MDR bacteria, this study was aimed to investigate the* in vitro* antibacterial activity as well as the antibiotic modulating activities of leaves of* Tristemma hirtum* and five other Cameroonian edible plants against selected Gram-negative MDR phenotypes.

## 2. Methods

### 2.1. Plant Material and Extraction

Six edible plants were collected in Bapa (5°16′0′′ north, 10°20′0′′ east) and Dschang (5°27′0′′ north, 10°4′0′′ east), two localities in the West Region of Cameroon. Samples collected were leaves of* Aframomum letestuanum* Gagnep. (Zingiberaceae) and* Tristemma hirtum* P. Beauv. (Melastomataceae), leaves and stem of* Aframomum alboviolaceum* (Ridl.) K.Schum. (Zingiberaceae), Pericarps of* Cucurbita pepo* Linn. (Cucurbitaceae) and* Raphia hookeri* Mann & Wendl. (Arecaceae), and the stem of* Physalis peruviana* L. (Solanaceae). The identification of the plants was done at the National Herbarium in Yaoundé (Cameroon), where the voucher specimens were conserved under the registration numbers (Table 1). The dried and powdered material (100 g) of each plant was macerated in 300 mL of methanol at room temperature for 48 h and then filtered using Whatman filter paper number 1. The filtrate obtained was concentrated using a rotary evaporator under reduced pressure to obtain the crude methanol extract, which was kept at 4°C until further use.

### 2.2. Chemicals

Six reference antibiotics (RA) purchased from Sigma-Aldrich (Saint-Quentin-Fallavier, France) were tested: ceftriaxone (CEF), chloramphenicol (CHL), ciprofloxacin (CIP), erythromycin (ERY), kanamycin (KAN), and tetracycline (TET);* p*-iodonitrotetrazolium chloride (INT) (Sigma-Aldrich) was used as bacterial growth revelator; dimethylsulfoxide (DMSO) was used to dissolve the plant extracts.

### 2.3. Bacteria, Culture Media, and Growth Conditions

The twenty strains of Gram-negative bacteria tested in this study were MDR isolates (laboratory collection) and reference strains (American Type Culture Collection) of* Escherichia coli* (ATCC8739, AG100A, AG100ATet, AG102, MC4100, and W3110),* Enterobacter aerogenes* (ATCC13048, CM64, EA27, EA289, and EA294),* Klebsiella pneumoniae* (ATCC11296, KP55, KP63, and K24),* Pseudomonas aeruginosa* (PA01 and PA124), and* Providencia stuartii* (NEA16, PS2636, and PS299645). The clinical strains were the laboratory collection from UMR-MD1, University of Marseille, France. The bacterial features are reported in Table S1 (see supplementary materials). The microorganisms were cultured overnight on* Mueller-Hinton Agar* (MHA) 24 h prior to any assay. The* Mueller-Hinton Broth* (MHB) was used as liquid culture medium for susceptibility tests.

### 2.4. Preliminary Phytochemical Screening

Potential classes of potential antibacterial phytochemicals such as alkaloids (Dragendorff's and Mayer's tests), terpenoids: sterols (Salkowski's test), saponins (foam test) and triterpenes (Liebermann-Burchard test), and phenolics: anthraquinones (Borntrager's test), flavonoids (aluminum chloride test), polyphenols (ferric chloride test), and tannins (gelatin test) (Table 2) were investigated according to the described phytochemical methods [19, 20].

### 2.5. Microbial Susceptibility Testing

The minimal inhibitory concentration (MIC) and minimum bactericidal concentration (MBC) determinations on bacteria were performed using the rapid INT colorimetric assay [21] with some modifications as previously described [16, 22]. Samples were dissolved in DMSO/MHB. The final concentration of DMSO was lower than 2.5%. The 2-fold dilutions of samples were made in 96-well microplates and the tested bacterial concentration was 1.5 × 10^6^ colony forming units (CFU)/mL. The microplates were incubated at 37°C for 18 h. All assays were in triplicate and were repeated thrice. Wells containing MHB, 100 *μ*L of inoculum, and DMSO to a final concentration of 2.5% served as negative control. Extracts and CHL were tested in the concentration ranges of 16–2048 *μ*g/mL and 2–256 *μ*g/mL, respectively. The MIC of each sample was detected after 18 h incubation at 37°C, following addition (40 *μ*L) of 0.2 mg/mL of INT and incubation at 37°C for 30 minutes as the lowest sample concentration that prevented the color change of the medium and exhibited complete inhibition of microbial growth [16, 22]. The MBC was determined by adding 50 *μ*L aliquots of the preparations which did not show any growth after incubation during MIC assays to 150 *μ*L of MHB. These preparations were further incubated at 37°C for 48 h. The MBC was regarded as the lowest concentration of a sample, which did not induce a color change after addition of INT as mentioned above [16, 22].

### 2.6. Antibiotic-Activity Modulation Assays

To evaluate the potentiating effect of the tested crude extracts, a preliminary assay was performed using the association of extracts at their various subinhibitory concentrations with antibiotics against one of a problematic bacterium,* P. aeruginosa* PA124. MIC/2 and MIC/4 of extracts were selected as the best subinhibitory concentrations [16, 23]. Samples were tested at various subinhibitory concentrations (MIC/2, MIC/4, MIC/8, and MIC/16). Results allowed selecting MIC/2 and MIC/4 as subinhibitory concentrations for further experiments on selected Gram-negative bacteria. Briefly, after serial dilution of antibiotic, extract was added to each well at its subinhibitory concentration and the bacterial inoculation was done; the MIC was further determined. Antibiotics were tested in the concentration ranges of 2–256 *μ*g/mL or 0.5–64 *μ*g/mL when it was necessary. Rows receiving antibiotic dilutions without extracts were used for the determination of the MICs of the antibiotics. The modulation factor was defined as the ratio of the MIC of antibiotic alone versus that of antibiotic in the presence of extract. Modulation factor ≥ 2 was set as the cut-off for biological significance of antibiotic-resistance modulating effects [24].

## 3. Results

### 3.1. Phytochemical Composition of Plant Extracts

The results of the qualitative phytochemical screening showed that each plant extract contains at least one of the secondary metabolites classes such as alkaloids, anthraquinones, flavonoids, phenols, saponins, tannins, steroids, and triterpenes. Polyphenols and steroids were present in all the extracts (Table 2).

### 3.2. Antibacterial Activity

Plant extracts were tested for their antibacterial activities on a panel of 20 Gram-negative bacteria. Results summarized in Table 3 showed that all extracts were active on at least one bacterial strain with MIC values ranging from 512 to 2048 *μ*g/mL. The extracts of* Raphia hookeri* pericarps (RHP),* Tristemma hirtum* leaves (THL), and* Cucurbita pepo* pericarps (CPP) were more active. They presented the lowest MIC value (512 *μ*g/mL) against* E. coli* ATCC8739 (RHP),* E. coli* AG100 (THL),* E. coli* MC4100 (CCP and THL),* P. stuartii *PS299645 (RHP, CCP, and THL),* E aerogenes *EA294 (RHP), and* P. aeruginosa *PA01 (RHP). In general, MBC values recorded for the extracts were more than 2048 *μ*g/mL.

### 3.3. Antibiotic-Resistance Modulation Activity of Extracts

The antibacterial activities of six commonly used antibiotics were evaluated in absence/presence of the different plant extracts. The results of the prescreening of the tested plant extracts for their antibiotic-resistance modulating effects against* P. aeruginosa *PA124 (Table 4) allowed us to select 4 plant extracts (leaves extracts of* T. hirtum*,* Aframomum letestuanum, Aframomum alboviolaceum, *and the pericarps extract of* Raphia hookeri*) for the study of their modulating effects against selected MDR bacteria, these at the concentrations equivalent to the half and quarter of their MIC values. It was observed that some extracts selectively improved the antibacterial activities of the tested antibiotics by decreasing their MIC values at about 2 to 64 times (Tables 5–8). The most important effects were observed with leaves extracts of* Tristemma hirtum *which significantly improved the antibacterial activities of all the tested antibiotics against 70% (7/10) to 100% (10/10) of the MDR bacteria used for the study. The activities of CHL, KAN, and CIP were mostly improved (2 to 128 times) as well as MIC/2 compared to at MIC/4 values (Table 8). The pericarp*s *extract of* Raphia hookeri* also showed significant antibiotic-potentiating activities in more than 70% (7/10) of the tested MDR bacteria. Its modulating effect was more important when it was associated with CHL, KAN, STP, ERY, and TET, mainly at the half of its MIC values (Table 7). The antibiotic-modulating effects of the other extracts (*Aframomum letestuanum *and* Aframomum alboviolaceum*) were not significant. They were observed on less than 50% of the tested MDR bacteria (Tables 5 and 6).

## 4. Discussion

### 4.1. Phytochemical Composition of Extracts

Plant secondary metabolites including flavonoids, phenols, terpenoids, steroids, saponins, and tannins are known for their antimicrobial activities [25–27]. In this study, phytochemical screening of the tested extracts indicated the presence of at least one of these metabolites in each extract (Table 2). Their presence in the studied extracts could therefore explain the observed activities. Also, terpenoids and phenolics were previously reported in* Aframomum alboviolaceum *[16],* Cucurbita pepo* [28, 29],* Physalis peruviana* [30],* Raphia hookeri* [31, 32], and* Tristemma hirtum* [33]. Their presence in the tested plant extract is in conformity with the previous phytochemical investigations.

### 4.2. Antibacterial Potential of Extracts

Based on the cut-off values indicating the antibacterial activity of plant extracts proposed by Kuete [34], many of the tested extracts, especially those from* Tristemma hirtum *leaves (THL),* Raphia hookeri *pericarps (RHP), and* Cucurbita pepo *pericarps (CPP) had weak (>512 *μ*g/mL) to no activity (MIC > 2048 *μ*g/mL) against the tested bacteria (Table 2). Previous studies have already demonstrated their* in vitro* antibacterial activities against many pathogenic microorganisms including bacteria. Oboh et al. [35] demonstrated the antibacterial properties of* Raphia hookeri* syrup against* E. coli*,* P. aeruginosa*, and* S. aureus*. According to these authors, the observed activity may be attributed to the presence of phenolic compounds including tannins, saponins, and flavonoids, which were also highlighted in the methanol extract used in this work. With regard to* Tristemma hirtum*, the studies carried out by Noumedem [36] previously demonstrated its antibacterial activity against selected sensitive bacterial strains including* E. coli*,* P. aeruginosa*,* S. flexneri, K. pneumonia, S. typhi, S. paratyphi, *and* E. faecalis.* Ahoua et al. [37] showed that a plant belonging to the same genus,* Tristemma coronatum*, presented inhibitory power against various bacterial strains. This is in accordance with the results obtained in this study. Noumedem et al. [17] showed that the methanol leaves extract of* Cucurbita pepo* has significant antibacterial activity against the bacterial strains used in this work. The difference of the activity may be due to the fact that different parts of the plants were used in the two studies. Even though the antibacterial activities of the tested plant extracts were in general moderate to low, if we consider the features of the bacteria used in this study (Table S1, supplementary materials) and the fact that the plants used are edible plants, some like the extract of leaves of* Tristemma hirtum *and extracts of pericarps of* Raphia hookeri *and* Cucurbita pepo* could be used to fight bacterial infections.

### 4.3. Antibiotic-Modulation Effects of Extracts

Infections due to MDR Gram-negative bacteria are particularly concerning as such bacteria are demonstrating resistance to practically all current licensed therapies [2, 4, 38]. Numerous ranges of novel approaches are under investigation as potential alternative treatments. Among them, the use of plant-derived substances to target different antibiotic resistance systems in the bacteria is considered as one of the best strategies [10, 39, 40]. In this study, nine methanol plant extracts were tested for their ability to modulate the activity of some common antibiotics. As the main results, it was observed that the activities of all tested antibiotics, especially those of CHL, KAN, CIP, and TET, were improved 2 to 128 times against more than 70% of the tested MDR bacteria in the presence of the extracts of leaves of* Tristemma hirtum* and pericarp*s *of* Raphia hookeri* (Tables 7 and 8). According to some reviews, plant-derived substances can act in synergy with antibiotics by inhibiting bacterial efflux pumps, allowing an increase of the intracellular concentrations of the antibiotics [7, 41]. Thus, because the bacteria used in this work express efflux pumps as the main resistance mechanism (Table S1, supplementary materials), the results obtained herein suggest that the aforesaid extracts could contain efflux pumps inhibitors [42]. According to Okusa and Duez [39], such effects may be due to the presence of alkaloids, flavonoids, terpenoids, and tannins in those extracts. Additionally, the tested extracts presented some antagonist actions with the antibiotics. This could be due to negative interactions between the antibiotics and the compounds present in the tested extracts. Finally, the most active extracts, extracts of leaves of* Tristemma hirtum* and pericarp*s *of* Raphia hookeri*, could be used to stimulate the renewed use of antibiotics with reduced effectiveness due to resistance.

## 5. Conclusion

With respect to the main findings of this work, the extracts of leaves of* Tristemma hirtum* and pericarps of* Raffia hookeri* appear as sources of substances that can be promising potentiating agents of antibiotics, even though they have moderate direct antibacterial action against the tested MDR bacteria.

## Figures and Tables

**Table 1:** Information on the studied plants.

Species (family); voucher number	Traditional uses	Bioactive or potentially bioactive components	Known antimicrobial activities of plants
* Aframomum alboviolaceum *(Ridl.) K.Schum. (Zingiberaceae); 34888/HNC	Against bacterial infections, hemorrhoid, and fever and as diuretic [16, 43, 44]	Alkaloids, flavonoids, phenols, triterpenes, and anthocyanin [16]	Methanol extract of rhizome against *Enterobacter aerogenes*, *Klebsiella pneumoniae*, *Enterobacter cloacae* [16], and *Haemonchus contortus* [45]

*Aframomum letestuanum* Gagnep. (Zingiberaceae); 43134/HNC	Against hemorrhage, muscular pains, nausea, and vomiting [46]	Not reported	Not reported

*Cucurbita pepo *Linn. (Cucurbitaceae); 42512/HNC	Against stomachache, for hepatoprotection, antidiabetic, antimicrobial, and anti-inflammatory hypertension [47–49].	Carotenoids, saponins, tannins, quinones, coumarins, lignins, and alkaloids [28, 29]	Ethanol extract of leaves against *Escherichia coli, Klebsiella pneumoniae, *and *providencia stuartii* [17]

* Physalis peruviana *L. (Solanaceae); 16547/SRF-Cam	Against asthma, hepatitis, malaria, and rheumatism [46]	Polyphenols and steroids [30]	Ethanol extract of seeds against *Lactococcus lactis *[50], ethanol extract and water extract of leaves and berries against *Listeria* spp. [51]

* Raphia hookeri* Mann & Wendl. (Arecaceae); 38372/HNC	As laxative and against malaria, measles, and infections [31, 52]	Alkaloids, flavonoids, phenols, tannins, and saponins [31, 32].	Effects of wine against* Escherichia coli, Pseudomonas aeruginosa, Bacillus cereus*, and* Staphylococcus aureus *[31, 35]

*Tristemma hirtum *P. Beauv. (Melastomataceae); 33936/HNC	Against typhoid fever, hemorrhoids, infertility, and skin diseases [33].	Alkaloids, flavonoids, polyphenols, steroids, tannins, saponins, and triterpenes [33]	Not reported

HNC: Herbier National du Cameroun; SRF-Cam: Société des Réserves Forestières du Cameroun.

**Table 2:** Extraction yields and phytochemical composition of the plant extracts.

Plant extracts	Part used	Yields (%)	alk	pol	flav	anthr	tan	tri	ster	sap
*Aframomum alboviolaceum*	Stem	3.1	+	+	+	+	+	+	+	−
	Leaves	8.7	+	+	+	+	−	+	+	+
*Aframomum letestuanum*	Leaves	3.7	+	+	+	−	+	+	+	+
*Cucurbita pepo*	Pericarps	9.1	+	+	+	−	+	+	+	−
*Physalis peruviana*	Stem	4.05	+	+	−	+	+	+	+	−
*Raphia hookeri*	Pericarps	5.2	+	+	+	+	+	−	+	+
*Tristemma hirtum*	Leaves	10.4	−	+	+	+	−	+	+	+

−: absent; +: present. Yield was calculated as the ratio of the mass of the obtained methanol extract/mass of the plant powder. alk: alkaloids; anthr: anthraquinones; flav: flavonoids; pol: polyphenols; sap: saponins; ster: steroids; tan: tannins; tri: triterpenes.

**Table 3:** Minimal inhibitory concentrations (MIC) and minimal bactericidal concentrations (MBC) of the plant extracts and chloramphenicol against bacteria strains.

Bacterial strains^a^	Samples^b^, MIC and MBC in *μ*g/mL (in brackets)							
	Plant extracts							Antibiotic
	AAS	AAL	ALL	CCP	PPS	RHP	THL	CHL
*Escherichia coli*								
ATCC8739	2048 (-)	2048 (-)	2048 (-)	2048 (-)	2048 (-)	512 (1024)	2048 (-)	**2** (8)
AG100A	1024 (2048)	1024 (-)	1024	512 (-)	2048 (-)	512 (1024)	512 (-)	32 (256)
AG100ATET	2048 (-)	1024 (-)	1024 (1024)	-	2048 (-)	1024 (-)	-	**8** (128)
AG102	-	-	-	-	-	-	-	32 (-)
W3110	1024 (-)	1024 (-)	1024 (1024)	1024 (-)	-	1024 (2048)	1024 (-)	**2** (8)
MC4100	1024 (-)	1024 (-)	1024 (-)	512 (-)	2048 (-)	1024 (-)	512 (-)	**2** (8)
*Enterobacter aerogenes*								
ATCC13048	1024 (-)	1024 (-)	1024 (-)	2048 (-)	2048 (-)	1024 (-)	2048 (-)	**2** (8)
EA289	-	-	-	2048 (-)	-	-	2048 (-)	128 (128)
EA294	1024 (2048)	1024 (2048)	1024 (2048)	2048 (-)	-	512 (-)	2048 (-)	**2** (8)
EA27	2048	-	-	2048 (-)	-	-	2048 (-)	64 (2)
CM64	-	-	-	-	-	-	-	16 (-)
*Klebsiella pneumoniae*								
ATCC11296	-	1024 (-)	1024 (-)	1024 (-)	-	-	1024 (-)	**8** (64)
KP55	1024 (-)	1024 (-)	1024 (-)	1024 (-)	-	512 (-)	1024 (-)	**2** (8)
KP63	-	-	-	-	-	-	-	64 (-)
K24	-	1024 (-)	1024 (-)	-	-	2048 (-)	-	**4** (256)
*Providencia stuartii*								
PS299645	2048 (-)	1024 (-)	1024 (-)	512 (-)	2048 (-)	512 (-)	512 (-)	32 (-)
NEA16	-	-	-	2048 (-)	-	-	2048 (-)	32 (-)
PS2636	-	-	-	1024 (-)	-	1024 (-)	1024 (-)	32 (-)
*Pseudomonas aeruginosa*								
PA01	-	1024 (-)	1024 (-)	1024 (-)	-	512 (-)	1024 (-)	128 (256)
PA124	-	-	-	-	-	1024 (-)	-	32 (-)

^a^Bacterial strain *[E.c: Escherichia coli, E.a: Enterobacter aerogenes, K.p: Klebsiella pneumoniae, P.s: Providencia stuartii, *and *P.a: Pseudomonas aeruginosa]*. ^b^Samples [AAS: *Aframomum alboviolaceum* (stem), AAL: *Aframomum alboviolaceum* (leaves), ALL: *Aframomum letestuanum* (leaves), CPP: *Cucurbita pepo* (pericarps), PPS: *Physalis peruviana* (stem), RHP: *Raphia hookeri* (pericarps), and THL: *Tristemma hirtum* (leaves); CHL: chloramphenicol]; MIC: minimal inhibitory concentration; MBC: minimal bactericidal concentration; -: MIC and MBC > 2048 *μ*g/mL; MIC in bold: significant activity.

**Table 4:** Preliminary evaluation of antibiotic-resistance modulatory activity of selected extracts at subinhibitory concentrations against *Pseudomonas aeruginosa *PA124.

Plant extracts	Extract concentration	MIC of antibiotic (*μ*g/mL) alone and in combination with extracts^a^ and fold increase of activity (in brackets)					
		CHL	KAN	CEF	CIP	TET	ERY
	0	128	128	128	64	16	128

AAL	MIC/2	32 **(4)**	64 **(2)**	128 (1)	64 (1)	8 **(2)**	128 (1)
	MIC/4	64 **(2)**	64 **(2)**	128 (1)	64 (1)	8 **(2)**	256 (0.5)
	MIC/8	64 **(2)**	128 (1)	128 (1)	64 (1)	16 (1)	256 (0.5)
	MIC/16	128 (1)	128 (1)	128 (1)	64 (1)	16 (1)	256 (0.5)

ALL	MIC/2	128 (1)	64 **(2)**	128 (1)	64 (1)	8 **(2)**	256 (0.5)
	MIC/4	128 (1)	64 **(2)**	128 (1)	64 (1)	8 **(2)**	256 (0.5)
	MIC/8	128 (1)	128 (1)	128 (1)	64 (1)	16 (1)	256 (0.5)
	MIC/16	128 (1)	128 (1)	128 (1)	64 (1)	16 (1)	256 (0.5)

RHP	MIC/2	32 **(4)**	32 **(4)**	64 **(2)**	32 **(2)**	8 **(2)**	64 **(2)**
	MIC/4	64 **(2)**	64 **(2)**	128 (1)	64 (1)	16 (1)	128 (1)
	MIC/8	128 (1)	64 **(2)**	256 (0.5)	64 (1)	16 (1)	128 (1)
	MIC/16	128 (1)	64 **(2)**	256 (0.5)	64 (1)	16 (1)	128 (1)

THL	MIC/2	8 **(16)**	64 **(2)**	128 (1)	32 **(2)**	8 **(2)**	128 (1)
	MIC/4	8 **(16)**	64 **(2)**	256 (0.5)	32 **(2)**	8 **(2)**	256 (0.5)
	MIC/8	32 **(4)**	64 **(2)**	256 (0.5)	32 **(2)**	8 **(2)**	256 (0.5)
	MIC/16	32 **(4)**	64 **(2)**	256 (0.5)	32 **(2)**	8 **(2)**	256 (0.5)

^a^Samples [AAL:* Aframomum alboviolaceum *(leaves), ALL: *Aframomum letestuanum *(leaves), RHP:* Raphia hookeri *(pericarps), THL: *Tristemma hirtum *(leaves), CHL: chloramphenicol, CEF: ceftriaxone, CIP: ciprofloxacin, ERY: erythromycin, KAN: kanamycin, STR: streptomycin, and TET: tetracycline]; in brackets: modulating factor; MIC: minimal inhibitory concentration. Values in bold represent modulating factor ≥ 2.

**Table 5:** Antibiotic-resistance modulatory activity of leaves extract of *Aframomum alboviolaceum*.

Antibiotics	Extract concentration	Bacteria, MIC (*μ*g/mL) and modulating factors (in brackets)							
		*Escherichia coli *		*Enterobacter aerogenes *	*Klebsiella pneumoniae *	*Providencia stuartii *	*Pseudomonas aeruginosa*		Modulating effect (%)
		AG100A	AG102	EA289	KP63	NEA16	PA01	PA124	
CHL	0	256	256	128	64	128	256	128	
	CMI/2	128 **(2)**	64 **(4)**	128 (1)	128 (0.5)	64 **(2)**	32 **(8)**	32 **(4)**	**71.42**
	CMI/4	128 **(2)**	128 **(2)**	256 (0.5)	128 (0.5)	128 (1)	128 **(2)**	64 **(2)**	42.85

KAN	0	8	64	64	64	64	64	128	
	CMI/2	8 (1)	32 **(2)**	256 (0.25)	128 (0.5)	32 **(2)**	32 **(4)**	64 **(2)**	57.14
	CMI/4	8 (1)	32 **(2)**	256 (0.25)	128 (0.5)	32 **(2)**	32 **(4)**	64 **(2)**	57.14

CEF	0	64	64	64	128	256	64	128	
	CMI/2	128 (0.5)	4 **(16)**	128 (0.5)	32 **(4)**	64 **(4)**	32 **(2)**	128 (1)	57.14
	CMI/4	128 (.05)	8 **(8)**	128 (0.5)	64 **(2)**	128 **(2)**	128 (0.5)	128 (1)	42.85

CIP	0	16	16	8	32	32	16	64	
	CMI/2	32 (0.5)	16 (1)	16 (0.5)	32 (1)	64 (0.5)	32 (0.5)	64 (1)	00.00
	CMI/4	64 (0.25)	16 (1)	16 (0.5)	64 (0.5)	64 (0.5)	32 (0.5)	64 (1)	00.00

TET	0	>64	64	32	64	32	>64	16	
	CMI/2	>64 (1)	64 (1)	32 (1)	>64 (1)	4 **(8)**	32 **(≥2)**	8 **(2)**	42.85
	CMI/4	>64 (1)	64 (1)	32 (1)	>64 (1)	8 **(4)**	>64 (1)	8 **(2)**	28.57

ERY	0	>256	128	>256	128	128	>256	128	
	CMI/2	>256 (1)	64 **(2)**	>256 (1)	128 (1)	16 **(8)**	64 **(≥4)**	128 (1)	42.85
	CMI/4	>256 (1)	64 **(2)**	>256 (1)	256 (0.5)	64 **(2)**	128 **(≥2)**	256 (0.5)	42.85

CHL: chloramphenicol, KAN: kanamycin, CEF: ceftriaxone, CIP: ciprofloxacin, TET: tetracycline, and ERY: erythromycin; in brackets: modulating factor; MIC: minimal inhibitory concentration. Values in bold represent modulating factor ≥ 2.

**Table 6:** Antibiotic-resistance modulatory activity of leaves extract of *Aframomum letestuanum*.

Antibiotics	Extract concentration	Bacteria, MIC (*μ*g/mL) and modulating factors (in brackets)							
		* Escherichia coli*		*Enterobacter aerogenes *	*Klebsiella pneumoniae *	*Providencia stuartii *	*Pseudomonas aeruginosa*		Modulating effect (%)
		AG100A	AG102	EA289	KP63	NEA16	PA01	PA124	
CHL	0	256	256	128	64	128	256	128	
	CMI/2	256 (1)	64 **(4)**	256 (0.5)	256 (0.25)	128 (1)	64 **(4)**	128 (1)	28.57
	CMI/4	256 (1)	256 (1)	256 (0.5)	256 (0.25)	128 (1)	128 **(2)**	128 (1)	14.28

KAN	0	8	64	64	64	64	64	128	
	CMI/2	32 (0.25)	64 (1)	64 (1)	128 (0.5)	128 (0.5)	32 **(2)**	64 **(2)**	28.57
	CMI/4	32 (0.25)	128 (0.5)	64 (1)	128 (0.5)	128 (0.5)	32 **(2)**	64 **(2)**	28.57

CEF	0	64	64	64	128	256	64	128	
	CMI/2	≤2 **(≥32)**	16 **(4)**	128 (0.5)	16 **(8)**	64 **(4)**	32 **(2)**	128 (1)	**71.42**
	CMI/4	64 (1)	32 **(2)**	256 (0.25)	32 **(4)**	64 **(4)**	128 (0.5)	128 (1)	42.85

CIP	0	16	16	8	32	32	16	64	
	CMI/2	16 (1)	16 (1)	32 (0.25)	64 (0.5)	64 (0.5)	32 (0.5)	64 (1)	00.00
	CMI/4	16 (1)	32 (0.5)	32 (0.25)	64 (0.5)	64 (0.5)	32 (0.5)	64 (1)	00.00

TET	0	>64	64	32	64	32	>64	16	
	CMI/2	>64 (1)	128 (0.5)	64 (0.5)	64 (1)	8 **(4)**	64 (1)	8 **(2)**	28.57
	CMI/4	>64 (1)	128 (0.5)	64 (0.5)	64 (1)	16 **(2)**	64 (1)	8 **(2)**	28.57

ERY	0	>256	128	>256	128	128	>256	128	
	CMI/2	>256 (1)	128 (0.5)	>256 (1)	128 (1)	32 **(4)**	128 **(≥2)**	256 (0.5)	28.57
	CMI/4	>256 (1)	128 (0.5)	>256 (1)	128 (1)	32 **(4)**	128 **(≥2)**	256 (0.5)	28.57

CHL: chloramphenicol, KAN: kanamycin, CEF: ceftriaxone, CIP: ciprofloxacin, TET: tetracycline, and ERY: erythromycin; in brackets: modulating factor; MIC: minimal inhibitory concentration. Values in bold represent modulating factor ≥ 2.

**Table 7:** Antibiotic-resistance modulatory activity of pericarps extract of *Raphia hookeri*.

Antibiotics	Extract concentration	Bacteria, MIC (*μ*g/mL) and modulating factors (in brackets)							
		*Escherichia coli*		*Enterobacter aerogenes*	*Klebsiella pneumoniae*	*Providencia stuartii*	*Pseudomonas aeruginosa*		Modulating effect (%)
		AG100A	AG102	EA289	KP63	NEA16	PA01	PA124	
CHL	0	256	256	128	64	128	256	128	
	CMI/2	128 **(2)**	32 **(8)**	32 **(4)**	32 **(2)**	16 **(8)**	128 **(2)**	32 **(4)**	**100.00**
	CMI/4	128 **(2)**	32 **(8)**	32 **(4)**	32 **(2)**	16 **(8)**	128 **(2)**	64 **(2)**	**100.00**

KAN	0	8	64	64	64	64	64	128	
	CMI/2	32 (0.25)	32 **(2)**	32 **(2)**	32 **(2)**	**32 (2)**	128 (1)	32 **(4)**	**71.42**
	CMI/4	32 (0.25)	32 **(2)**	32 **(2)**	32 **(2)**	**32 (2)**	128 (1)	64 **(2)**	**71.42**

CEF	0	64	64	64	128	256	64	128	
	CMI/2	64 (1)	4 **(32)**	64 (1)	32 **(4)**	64 **(4)**	32 **(2)**	64 **(2)**	**71.42**
	CMI/4	64 (1)	4 **(32)**	64 (1)	32 **(4)**	64 **(4)**	32 **(2)**	128 (1)	57.14

CIP	0	16	16	8	32	32	16	64	
	CMI/2	8 **(2)**	32 (0.5)	16 (0.5)	2 **(16)**	64 (0.5)	16 (1)	32 **(2)**	42.85
	CMI/4	8 **(2)**	32 (0.5)	16 (0.5)	2 **(16)**	64 (0.5)	16 (1)	64 (1)	28.57

TET	0	>64	64	32	64	32	>64	16	
	CMI/2	>64 (1)	64 (1)	8 **(4)**	32 **(2)**	4 **(8)**	64 **(≥2)**	8 **(2)**	**71.42**
	CMI/4	>64 (1)	64 (1)	8 **(4)**	32 **(2)**	4 **(8)**	64 **(≥2)**	16 (1)	57.14

ERY	0	>256	128	>256	128	128	>256	128	
	CMI/2	>256 (1)	256 (0.5)	128 **(2)**	64 **(2)**	32 **(4)**	128 **(≥2)**	64 **(2)**	**71.42**
	CMI/4	>256 (1)	256 (0.5)	>256 (1)	64 **(2)**	32 **(4)**	128 **(≥2)**	128 (1)	42.85

CHL: chloramphenicol, KAN: kanamycin, CEF: ceftriaxone, CIP: ciprofloxacin, TET: tetracycline, and ERY: erythromycin; in brackets: modulating factor; MIC: minimal inhibitory concentration. Values in bold represent modulating factor ≥ 2.

**Table 8:** Antibiotic-resistance modulatory activity of leaves extract of *Tristemma hirtum*.

Antibiotics	Extract concentration	Bacteria, MIC (*μ*g/mL) and modulating factors (in brackets)							
		* Escherichia coli*		*Enterobacter aerogenes*	*Klebsiella pneumoniae*	*Providencia stuartii *	*Pseudomonas aeruginosa*		Modulating effect (%)
		AG100A	AG102	EA289	KP63	NEA16	PA01	PA124	
CHL	0	256	256	128	64	128	256	128	
	CMI/2	128 **(2)**	≤2 **(≥128)**	16 **(8)**	16 **(4)**	16 **(8)**	64 **(4)**	8 **(16)**	**100.00**
	CMI/4	128 **(2)**	≤2 **(≥128)**	16 **(8)**	16 **(4)**	16 **(8)**	64 **(4)**	8 **(16)**	**100.00**

KAN	0	8	64	64	64	64	64	128	
	CMI/2	≤2 **(≥4)**	16 **(4)**	≤2 **(≥32)**	16 **(4)**	16 **(4)**	≤2 **(≥64)**	64 **(2)**	**100.00**
	CMI/4	≤2 **(≥4)**	16 **(4)**	≤2 **(≥32)**	32 **(2)**	32 **(2)**	≤2 **(≥64)**	64 **(2)**	**100.00**

CEF	0	64	64	64	128	256	64	128	
	CMI/2	8 **(8)**	8 **(8)**	8 **(8)**	16 **(8)**	32 **(8)**	≤2 **(≥32)**	128 (1)	**85.71**
	CMI/4	128 (0.5)	32 **(2)**	32 **(2)**	32 **(4)**	32 **(8)**	≤2 **(≥32)**	256 (0.5)	**71.42**

CIP	0	16	16	8	32	32	16	64	
	CMI/2	8 **(2)**	≤0.5 **(≥32)**	≤0.5 **(≥16)**	4 **(8)**	16 **(2)**	≤0.5 **(≥32)**	32 **(2)**	**100.00**
	CMI/4	8 **(2)**	4 **(8)**	≤0.5 **(≥16)**	4 **(8)**	16 **(2)**	≤0.5 (≥**32**)	32 **(2)**	**100.00**

TET	0	>64	64	32	64	32	>64	16	
	CMI/2	32 **(≥2)**	32 **(2)**	≤0.5 **(≥64)**	4 **(16)**	4 **(8)**	32 **(≥4)**	8 **(2)**	**100.00**
	CMI/4	32 **(≥2)**	32 **(2)**	8 **(4)**	8 **(8)**	4 **(8)**	64 **(≥2)**	8 **(2)**	**100.00**

ERY	0	>256	128	>256	128	128	>256	128	
	CMI/2	128 **(≥2)**	8 **(16)**	16 **(≥16)**	32 **(4)**	64 **(2)**	32 **(≥8)**	128 (1)	**85.71**
	CMI/4	128 **(≥2)**	16 **(8)**	32 **(≥8)**	32 **(4)**	64 **(2)**	64 **(≥4)**	256 (0.5)	**85.71**

CHL: chloramphenicol, KAN: kanamycin, CEF: ceftriaxone, CIP: ciprofloxacin, TET: tetracycline, and ERY: erythromycin; in brackets: modulating factor; MIC: minimal inhibitory concentration. Values in bold represent modulating factor ≥ 2.

## Supplementary Materials

Supplementary Materials Table  S1: Gram-negative bacteria used and their features and further details on the antibiotic-resistance profiles of tested Gram-negative bacteria.

## References

[1] Bassetti M., Ginocchio F., Mikulska M., Taramasso L., Giacobbe D. R. (2011). Will new antimicrobials overcome resistance among Gram-negatives?. *Expert Review of Anti-infective Therapy*.

[2] Xu Z.-Q., Flavin M. T., Flavin J. (2014). Combating multidrug-resistant Gram-negative bacterial infections. *Expert Opinion on Investigational Drugs*.

[3] Bassetti M., Pecori D., Peghin M. (2016). Multidrug-resistant Gram-negative bacteria-resistant infections: Epidemiology, clinical issues and therapeutic options. *Italian Journal of Medicine*.

[4] Huwaitat R., McCloskey A. P., Gilmore B. F., Laverty G. (2016). Potential strategies for the eradication of multidrug-resistant Gram-negative bacterial infections. *Future Microbiology*.

[5] Kamicker B. J., Sweeney M. T., Kaczmarek F. (2008). Bacteria efflux pomp inhibitors. *Methods in molecular medicine*.

[6] Zilberberg M. D., Shorr A. F., Micek S. T., Vazquez-Guillamet C., Kollef M. H. (2014). Multi-drug resistance, inappropriate initial antibiotic therapy and mortality in Gram-negative severe sepsis and septic shock: a retrospective cohort study. *Critical Care*.

[7] Stavri M., Piddock L. J. V., Gibbons S. (2007). Bacterial efflux pump inhibitors from natural sources. *Journal of Antimicrobial Chemotherapy*.

[8] Liscio J. L., Mahoney M. V., Hirsch E. B. (2015). Ceftolozane/tazobactam and ceftazidime/avibactam: Two novel *β*-lactam/*β*-lactamase inhibitor combination agents for the treatment of resistant Gram-negative bacterial infections. *International Journal of Antimicrobial Agents*.

[9] Zhanel G. G., Chung P., Adam H. (2014). Ceftolozane/tazobactam: A novel cephalosporin/*β*-lactamase inhibitor combination with activity against multidrug-resistant gram-negative bacilli. *Drugs*.

[10] Sibanda T., Okoh A. I. (2007). The challenges of overcoming antibiotic resistance: plant extracts as potential sources of antimicrobial and resistance modifying agents. *African Journal of Biotechnology*.

[11] Aiyegoro O. A., Okoh A. I. (2009). Use of bioactive plant products in combination with standard antibiotics: implications in antimicrobial chemotherapy. *Journal of Medicinal Plants Research*.

[12] Saleem M., Nazir M., Ali M. S. (2010). Antimicrobial natural products: an update on future antibiotic drug candidates. *Natural Product Reports*.

[13] Fankam A. G., Kuete V., Voukeng I. K., Kuiate J. R., Pages J.-M. (2011). Antibacterial activities of selected Cameroonian spices and their synergistic effects with antibiotics against multidrug-resistant phenotypes. *BMC Complementary and Alternative Medicine*.

[14] Lacmata S. T., Kuete V., Dzoyem J. P. (2012). Antibacterial activities of selected cameroonian plants and their synergistic effects with antibiotics against bacteria expressing MDR phenotypes. *Evidence-Based Complementary and Alternative Medicine*.

[15] Voukeng I. K., Kuete V., Dzoyem J. P. (2012). Antibacterial and antibiotic-potentiation activities of the methanol extract of some cameroonian spices against Gram-negative multi-drug resistant phenotypes. *BMC Research Notes*.

[16] Djeussi D. E., Noumedem J. A. K., Seukep J. A. (2013). Antibacterial activities of selected edible plants extracts against multidrug-resistant Gram-negative bacteria. *BMC Complementary and Alternative Medicine*.

[17] Noumedem J. A. K., Mihasan M., Lacmata S. T., Stefan M., Kuiate J. R., Kuete V. (2013). Antibacterial activities of the methanol extracts of ten Cameroonian vegetables against Gram-negative multidrug-resistant bacteria. *BMC Complementary and Alternative Medicine*.

[18] Dzotam J. K., Kuete V. (2017). Antibacterial and Antibiotic-Modifying Activity of Methanol Extracts from Six Cameroonian Food Plants against Multidrug-Resistant Enteric Bacteria. *BioMed Research International*.

[19] Harbone J. B. (1973). *Phytochemical Methods: A Guide to Modern Techniques of Plant Analysis*.

[20] Kuete V. (2013). *Medicinal Plant Research in Africa in: Pharmacology and Chemistry*.

[21] Eloff J. N. (1998). A sensitive and quick microplate method to determine the minimal inhibitory concentration of plant extracts for bacteria. *Planta Medica*.

[22] Kuete V., Wabo G. F., Ngameni B. (2007). Antimicrobial activity of the methanolic extract, fractions and compounds from the stem bark of Irvingia gabonensis (Ixonanthaceae). *Journal of Ethnopharmacology*.

[23] Fankam A. G., Kuiate J.-R., Kuete V. (2017). Antibacterial and antibiotic resistance modulatory activities of leaves and bark extracts of Recinodindron heudelotii (Euphorbiaceae) against multidrug-resistant Gram-negative bacteria. *BMC Complementary and Alternative Medicine*.

[24] Kovač J., Gavarić N., Bucar F., Možina S. S. (2014). Antimicrobial and resistance modulatory activity of alpinia katsumadai seed phenolic extract, essential oil and post-distillation extract. *ood Technology and Biotechnology*.

[25] Cowan M. M. (1999). Plant products as antimicrobial agents. *Clinical Microbiology Reviews*.

[26] Ríos J. L., Recio M. C. (2005). Medicinal plants and antimicrobial activity. *Journal of Ethnopharmacology*.

[27] Compean K. L., Ynalvez R. A. (2014). Antimicrobial activity of plant secondary metabolites: A review. *Research Journal of Medicinal Plant*.

[28] CARBIN B. ‐., LARSSON B., LINDAHL O. (1990). Treatment of Benign Prostatic Hyperplasia with Phytosterols. *British Journal of Urology*.

[29] Karpagam T., Varalakshmi B., Bai J. S., Gomathi S. (2011). Effect of different doses of Cucurbita pepo linn extract as an anti-Inflammatory and analgesic nutraceautical agent on inflamed rats. *International journal of periodontics & restorative dentistry*.

[30] Ramadan M. F., Morsel J.-T. (2003). Oil goldenberry (Physalis peruviana L.). *Journal of Agricultural and Food Chemistry*.

[31] Ibegbulem C. O., Alisi C. S., Nwankpa P., Amadi B. A., Agiang M. A., Ekam V. S. (2012). Medicinal values of Elaeis guineensisand Raphia hookeriwines. *Journal of Research in Biology*.

[32] Ogbuagu M. N. (2008). Vitamins, phytochemicals and toxic elements in the pulp and seed of raphia palm fruit (Raphia hookeri). *Fruits*.

[33] Dongmo M. R. C. (2009). *Evaluation of antidermatophytic activity of methanol extracts and fractions of Acalypha manniana (Euphorbiaceae) and Tristemma hirtum (Melastomataceae) [M.S. thesis]*.

[34] Kuete V. (2010). Potential of Cameroonian plants and derived products against microbial infections: A review. *Planta Medica*.

[35] Oboh O. F., Iyare L., Idemudia M., Enabulele S. (2016). Physico-chemical and nutritional characteristics, and antimicrobial activity of oil palm syrup, raffia palm syrup and honey. *IOSR Journal of Pharmacy and Biological Sciences*.

[36] Noumedem J. A. K. (2009). *Antimicrobial, antioxidant and ant-diarrhea properties of Acalypha manniana and Tristemma hirtum [M.s. thesis]*.

[37] Ahoua A. R. C., Konan A. G., Bonfoh B., Koné M. W. (2015). Antimicrobial potential of 27 plants consumed by chimpanzees (Pan troglodytes verus Blumenbach) in Ivory Coast. *BMC Complementary and Alternative Medicine*.

[38] Mascellino M. T., Angelis M. D., Oliva A. (2017). Multi-drug resistant gram-negative bacteria: antibiotic-resistance and new treatment strategies. *Diagnostic Pathology: Open Access*.

[39] Okusa P. N., Duez P., Varela A., Ibañez J. (2009). Chapitre 13: Medicinal plants: a tool to overcome antibiotic resistance?. *Medicinal Plants: Classification, Biosynthesis and Pharmacology*.

[40] Matias E. F. F., Santos K. K. A., Almeida T. S., Costa J. G. M., Coutinho H. D. M. (2010). Enhancement of antibiotic activity by Cordia verbenacea DC. *Latin American Journal of Pharmacy*.

[41] Tegos G., Stermitz F. R., Lomovskaya O., Lewis K. (2002). Multidrug pump inhibitors uncover remarkable activity of plant antimicrobials. *Antimicrobial Agents and Chemotherapy*.

[42] Braga L. C., Leite A. A. M., Xavier K. G. S. (2005). Synergic interaction between pomegranate extract and antibiotics against *Staphylococcus aureus*. *Canadian Journal of Microbiology*.

[43] Abreu P. M., Noronha R. G. (1997). Volatile constituents of the rhizomes of Aframomum alboviolaceum (Ridley) K. Schum. from Guinea-Bissan. *Flavour and Fragrance Journal*.

[44] Ilumbe G. B., Van Damme P., Lukoki F. L., Joiris V., Visser M., Lejoly J. (2014). Contribution à létude des plantes médicinales dans le traitement des hémorroides par les pygmées Twa et leur voisin Oto de Bikoro, en RDC. *Congo Sciences*.

[45] Koné W. M., Atindehou K. K., Dossahoua T., Betschart B. (2005). Anthelmintic activity of medicinal plants used in northern Côte d'Ivoire against intestinal helminthiasis. *Pharmaceutical Biology*.

[46] Yemele D. M., Telefo B. P., Goka S. C. (2015). In vtro cytotoxicity studies of sixteen plants used for pregnant women's health conditions in menoua division-West Cameroon. *International Journal of Phytomedicine*.

[47] Adepoju G. K. A., Adebanjo A. A. (2011). Effect of consumption of Cucurbita pepo seeds on haematological and biochemical parameters. *African Journal of Pharmacy and Pharmacology*.

[48] Jafarian A., Zolfaghari B., Parnianifard M. (2012). The effects of methanolic, chloroform, and ethylacetate extracts of the cucurbita pepo L. On the delay type hypersensitivity and antibody production. *Research in Pharmaceutical Sciences*.

[49] Sarkar S., Guha D. (2008). Effect of ripe fruit pulp extract of Cucurbita pepo Linn. in aspirin induced gastric and duodenal ulcer in rats. *Indian Journal of Experimental Biology (IJEB)*.

[50] Çakir Ö., Pekmez M., Çepni E., Candar B., Fidan K. (2014). Evaluation of biological activities of physalis peruviana ethanol extracts and expression of Bcl-2 genes in HeLa cells. *Food Science and Technology*.

[51] Cueva M. B., Tigre León R. A., Angel Yanchaliquín M. M., Herminia Sanaguano Salguero I. F. (2017). Antibacterial Effects of Uvilla (Physalis peruviana L.) extracts against Listeria spp. Isolated from Meat in Ecuador. *International Journal of Current Microbiology and Applied Sciences*.

[52] Irvine R. (1961). *Woody Plant of Ghana with Special Reference to Their Uses*.

