# Supplementary material for: Tristemma hirtum and Five Other Cameroonian Edible Plants with Weak or No Antibacterial Effects Modulate the Activities of Antibiotics against Gram-Negative Multidrug-Resistant Phenotypes
Source: ScientificWorldJournal. 2018 Mar 22;2018:7651482. doi: 10.1155/2018/7651482 (PMC5885400; doi:10.1155/2018/7651482)
Supplement: Supplementary Materials — Table S1: Gram-negative bacteria used and their features and further details on the antibiotic-resistance profiles of tested Gram-negative bacteria. [file 7651482.f1.doc]

***Tristemma hirtum* and five other Cameroonian edible plants with weak or no anti-bacterial effects modulate the activities of antibiotics against Gram-negative multi-drug resistant phenotypes**

Gaëlle S. Nguenang^1^, Armelle T. Mbaveng**^1^**, Aimé G. Fankam**^1^**, Hermione T. Manekeng^1^, Paul Nayim^1^, Brice E. N. Wamba^1^, and Victor Kuete**^1*^**

*^1^Department of Biochemistry, Faculty of Science, University of Dschang, Cameroon*

**Author’s addresses**

*Gaëlle S. Nguenang:* [*sgaelle78@yahoo.com*](mailto:sgaelle78@yahoo.com)

*Armelle T. Mbaveng:* [*armkuete@yahoo.fr*](mailto:armkuete@yahoo.fr)

*Aimé G. Fankam;* [*agfankam@yahoo.fr*](mailto:agfankam@yahoo.fr)

*Hermione T. Manekeng:* [*hermionemanekeng@yahoo.fr*](mailto:hermionemanekeng@yahoo.fr)

*Paul Nayim:* [*nayimpaul@yahoo.fr*](mailto:nayimpaul@yahoo.fr)

*Brice E. N. Wamba:* [*wambaelvis@yahoo.fr*](mailto:wambaelvis@yahoo.fr)

*Victor Kuete:* [*kuetevictor@yahoo.fr*](mailto:kuetevictor@yahoo.fr)

***Corresponding authors:***

*∗ Tel.: +237 677355927; E-mail address: kuetevictor@yahoo.fr (Prof. Dr Victor Kuete)*

**Table S1. Gram-negative bacteria used and their features**

| **Strains** | **Features** | **References** |
| --- | --- | --- |
| ***Escherichia coli*** |  |  |
| ATTC 8739 | Reference strain |  |
| AG 100 | Wild-type *E. coli* K-12 expressing *Acr AB* efflux pumps | [1] |
| AG 100ATet | ΔacrAB mutant AG 100A Tet^r^ | [1] |
| AG 102 | AG 100 expressing *Acr AB* pumps | [2] |
| W 3110 | Wild-type E.*coli* K-12 | [3] |
| MC4100 | Wild-type E. coli K-12, KAN^R^ expressing *ABC* pump | [3] |
| ***Enterobacter aerogenes*** |  |  |
| ATCC 13048 | Reference strain |  |
| EA3 | Clinical MDR isolate CHL^R^, NOR^R^,  MOX^R^, CFT^R^, ATM^R^, FEP^R^ | [4] |
| EA 27 | Clinical MDR isolate exhibiting  energy-dependent norfloxacin and  chloramphenicol efflux with KAN^R^ and  AMP^R^ and NAL^R^ and STR^R^ and TET^R^ | [5, 6] |
| EA 289 | KAN sensitive derivative of EA27 | [4] |
| EA294 | EA289 expressing *AcrA* pump having KAN^r^ | [4, 7] |
| EA 298 | EA 289 tolC:KAN^R^ | [4, 7] |
| ***Klebsiella pneumoniae*** |  |  |
| ATCC11296 | Reference strain |  |
| K 24 | Clinical MDR isolate *AcrAB-Tolc* | Clinical laboratory collection of UMR-MD1, University of Marseille, France |
| Kp 55 | Clinical MDR isolate, TET^R^, AMP^R^, ATM^R^, and CEF^R^ | [8] |
| Kp 63 | Clinical MDR isolate, TET^R^, CHL^R^, AMP^R^, and ATM^R^ | [8] |
| ***Providencia stuartii*** |  |  |
| NEA 16 | Clinical MDR isolate, *AcrAB-TolC* | [9] |
| PS299645 | Clinical MDR isolate, *AcrAB-TolC* |
| PS2636 | Clinical MDR isolate, *AcrAB-TolC* |
| ***Pseudomonas aeruginosa*** |  |  |
| PA 01 | Reference strain |  |
| PA 124 | Clinical MDR isolate | [10] |

^a^AMP^R^, ATM^R^, CEF^R^, CFT^R^, CHL^R^,CIP^R^, ERM^R^, FEP^R^, FLX^R^, IM/CS^R^, KAN^R^, MOX^R^, OFX^R^, STR^R^, TET^R^, Resistancetoampicillin, aztreonam, cephalothin, cefadroxil, chloramphenicol, Ciprofloxacin, Erythromycin, cefepime,Flomoxef, Imipenem/ Cilastatinsodium, kanamycin, moxalactam, streptomycin, andtetracycline; MDR : Multidrugresistant.

**References.**

[1] M. Viveiros, A. Jesus, M. Brito et al., “Inducement and reversal of tetracycline resistance in *Escherichia coli* K-12 and expression of proton gradient-dependent multidrug efflux pump genes,” Antimicrobial Agents and Chemotherapy, vol. 49, no. 8, pp. 3578–3582, 2005.

[2] C. A. Elkins and L. B. Mullis, “Substrate competition studies using whole-cell accumulation assays with the major tripartite multidrug efflux pumps of *Escherichia coli*,” *Antimicrobial Agents and Chemotherapy*, vol. 51, no. 3, pp. 923–929, 2007.

[3] P. Baglioni, L. Bini, S. Liberatori, V. Pallini, and L. Marri, “Proteome analysis of *Escherichia coli* W3110 expressing an heterologous sigma factor,” *Proteomics*, vol. 3, no. 6, pp. 1060–1065, 2003.

[4] D. Ghisalberti, M. Masi, J. M. Pages, and J. Chevalier, “Chloramphenicol and expression of multidrug efflux pump in *Enterobacter aerogenes*,” *Biochemical and Biophysical Research*

*Communications*, vol. 328, no. 4, pp. 1113–1118, 2005.

[5] M. Mallea, A. Mahamoud, J. Chevalier et al., “Alkylamino- quinolines inhibit the bacterial antibiotic efflux pump in multidrug-resistant clinical isolates,” *Biochemical Journal*, vol. 376, no. 3, pp. 801–805, 2003.

[6] M. Mallea, J. Chevalier, C. Bornet et al., “Porin alteration and active efflux: two *in vivo* drug resistance strategies used by *Enterobacter aerogenes*,” *Microbiology*, vol. 144, no. 11, pp. 3003–3009, 1998.

[7] E. Pradel and J. M. Pages, “The AcrAB-TolC efflux pump contributes to multidrug resistance in the nosocomial pathogen *Enterobacter aerogenes*,” Antimicrobial Agents and Chemotherapy, vol. 46, no. 8, pp. 2640–2643, 2002.

[8] J. Chevalier, J. M. Pages, A. Eyraud, and M. Mallea, “Membrane permeability modifications are involved in antibiotic resistance in *Klebsiella pneumoniae*,” *Biochemical and Biophysical Research Communications*, vol. 274, no. 2, pp. 496–499, 2000.

[9] Q. T. Tran, K. R. Mahendran, E. Hajjar et al., “Implication of porins in β-lactam resistance of *Providencia stuartii*,” *Journal of Biological Chemistry*, vol. 285, no. 42, pp. 32273–32281, 2010.

[10] V. Lorenzi, A. Muselli, A. F. Bernardini et al., “Geraniol restores antibiotic activities against multidrug-resistant isolates from gram-negative species,” *Antimicrobial Agents and Chemotherapy*, vol. 53, no. 5, pp. 2209–2211, 2009.
